# Sol-Gel Graphene Oxide-Coated Fabric Disks as Sorbents for the Automatic Sequential-Injection Column Preconcentration for Toxic Metal Determination in Distilled Spirit Drinks

**DOI:** 10.3390/molecules28052103

**Published:** 2023-02-23

**Authors:** Natalia Manousi, Abuzar Kabir, Kenneth G. Furton, Aristidis Anthemidis

**Affiliations:** 1 Laboratory of Analytical Chemistry, Department of Chemistry, Aristotle University of Thessaloniki, 54124 Thessaloniki, Greece; 2 International Forensic Research Institute, Department of Chemistry and Biochemistry, Florida International University, Miami, FL 33131, USA

**Keywords:** fabric disk sorptive extraction, sol-gel graphene oxide, sequential injection, atomic absorption spectrometry, metals, alcoholic beverages

## Abstract

Sol-gel graphene oxide-coated polyester fabric platforms were synthesized and used for the on-line sequential injection fabric disk sorptive extraction (SI-FDSE) of toxic (i.e., Cd(II), Cu(II) and Pb(II)) metals in different distilled spirit drinks prior to their determination by electrothermal atomic absorption spectrometry (ETAAS). The main parameters that could potentially influence the extraction efficiency of the automatic on-line column preconcentration system were optimized and the SI-FDSE-ETAAS method was validated. Under optimum conditions, enhancement factors of 38, 120 and 85 were achieved for Cd(II), Cu(II) and Pb(II), respectively. Method precision (in terms of relative standard deviation) was lower than 2.9% for all analytes. The limits of detection for Cd(II), Cu(II) and Pb(II) were 1.9, 7.1 and 17.3 ng L^−1^, respectively. As a proof of concept, the proposed protocol was employed for the monitoring of Cd(II), Cu(II), and Pb(II) in distilled spirit drinks of different types.

## 1. Introduction

A wide variety of metals and metalloids have been detected in alcoholic beverages, (e.g., As, Cd, Cr, Co, Cu, Fe, Mn, Ni, Sn, Pb, and Zn), the presence of which can be attributed to the raw materials used for their production; to the substances added during brewing; and to the equipment used during the storage, bottling, distillation and aging process [1,2]. Although elements such as Cu are essential for living organisms, long-term exposure to high concentrations can lead to chronic toxicosis [2]. On the other hand, metals such as Cd and Pb are considered to be highly toxic even at low concentration levels and they are associated with neurological disorders, anemia, kidney damage, liver damage and gastrointestinal damage [3,4]. Besides the impact of metals in human health, their presence in alcoholic beverages can significantly influence the quality of the product [1]. Depending on the type of the alcoholic beverage, a very wide range of metal concentration can be observed. Cd concentration can range from not detected up to 5.31 mg L^−1^, Pb concentration can range from not detected up to 1.125 mg L^−1^ and Cu concentration can range from not detected up to 14.6 mg L^−1^ [1].

Undoubtedly, electro-thermal atomic absorption spectrometry (ETAAS) is a powerful analytical spectrometric technique for metal determination due to its excellent applicability, high sensitivity, reduced operational cost and method simplicity [5,6,7]. However, due to the low concentration of the trace elements in the distilled spirit samples, a preconcentration step is necessary for the sensitive and accurate metal determination in complex samples. Currently, a lot of attention is being paid to the development of on-line automated sample preparation systems as an alternative to batch-mode extraction procedures. According to the principles of green sample preparation that were recently introduced [8], the development of automatic sample preparation procedures can contribute to a reduction in the required amount of chemicals and waste generated while providing a larger sample throughput. At the same time, automation can also assist in the minimization of the operator’s exposure to hazardous chemicals, thus reducing the risk of accidents. Although there are various on-line approaches for the extraction and preconcentration of metals, most of them focused on environmental and biological samples, and on-line systems for the monitoring of the metal content of alcoholic beverages are limited [9,10].

Fabric phase sorptive extraction (FPSE) is an evolutionary sample preparation technique that utilizes sol-gel organic–inorganic sorbent-coated fabric substrates with high thermal, chemical and solvent stability as extraction platforms [11]. A plethora of sol-gel coatings can be easily prepared, resulting in the fabrication of neutral, anion and cation exchangers, zwitterionic, and mixed mode zwitterionic sorptive phases [12]. FPSE has proved to be a significant sample preparation technique for the determination of a wide variety of organic and inorganic chemical species at trace- and ultra-trace-level concentrations [11,13]. Recently, the on-line flow injection–fabric disk sorptive extraction (FI-FDSE) technique was proposed as an automatic alternative to FPSE [14]. This technique offers various benefits such as high extraction efficiency, good reproducibility and sensitivity, and sorbent reusability [14,15]. Sol-gel sorbents have proved to be an important tool in environmental analysis [16], food analysis [17] and bioanalysis [18].

The second generation of flow injection techniques, known as sequential injection analysis (SIA), is based on the usage of programmable, bi-directional discontinuous flow, which is precisely coordinated and controlled by a computer. Due to its inherent characteristics, SIA exhibits multiple benefits including low chemical consumption, versatility, manifold simplicity, low operational cost, and low waste generation [19,20,21]. Although the FI-FDSE technique is a powerful analytical technique for on-line metal determination [14,15], its operation in on-line sequential injection (SI) mode has not yet been reported.

In this work, novel sol-gel graphene oxide-coated polyester FDSE membranes were synthesized and used to prepare a reusable and renewable on-line fabric disk sorbent extraction microcolumn. Graphene oxide has been proved to be a powerful material for the extraction of metals [22,23]. The proposed platform was employed for the first time for the effective preconcentration and determination of Cd(II), Cu(II) and Pb(II) as model analytes in distilled spirit drinks as a front end to ETAAS. The optimization of the main factors of the FDSE procedure was conducted to ensure the high extraction efficiency of the target analytes. The herein-developed protocol was validated and used for analysis of real samples.

## 2. Results and Discussion

### 2.1. Characterization of the Sol-Gel Graphene Oxide-Coated Polyester Fabric Membranes

**Figure 1:**
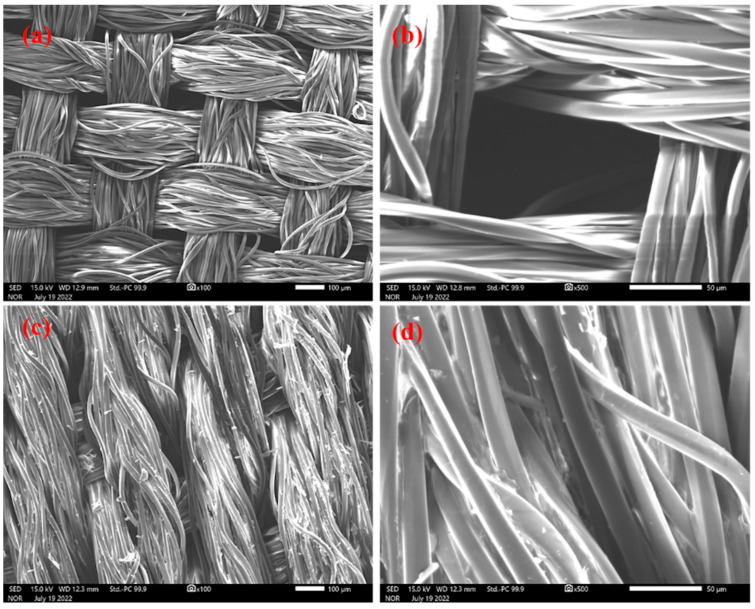
Scanning electron microscopy images of (**a**) uncoated polyester fabric at 100× magnifications; (**b**) demonstration of built-in pores in polyester fabric at 500× magnifications; (**c**) sol-gel graphene oxide-coated polyester FPSE membrane at 100× magnifications; (**d**) demonstration of the uniformity of the sol-gel graphene oxide coating at 500× magnifications.

The sol-gel graphene oxide-coated FPSE membranes were characterized by Fourier Transform Infrared Spectroscopy (FT-IR) and Scanning Electron Microscopy (SEM). FT-IR spectra provides important information about the functional makeup of the building blocks as well as the information regarding successful integration of the building blocks in the final product and its functional composition (Supplementary Materials, Figures S1–S3). Moreover, the SEM images shed light on the surface morphology of the fabric substrate and coated sol-gel sorbent. The scanning electron micrographs (SEM) images of (a) uncoated polyester fabric at 100× magnifications; (b) built-in pores in polyester fabric at 500× magnifications; (c) sol-gel graphene oxide-coated polyester FPSE membrane at 100× magnifications; (d) uniformity of the sol-gel graphene oxide coating at 500× magnifications are shown in Figure 1. The sol-gel coatings are uniformly distributed on the polyester substrate. The through pores of the polyester fabric remained intact even after the sol-gel sorbent coating, allowing for the rapid permeation of aqueous sample through the FPSE membrane bed during the analyte extraction. The rapid permeation of the sample through the extraction bed facilitates fast extraction kinetic.

### 2.2. Optimization of the On-Line Column Preconcentration Procedure

The main hydrodynamic and chemical factors of the SI-FDSE procedure were optimized by means of the one-variable-at-a-time approach (OVAT). In this case, each factor is individually examined, while the other factors remain constant. Method optimization was performed using aqueous standard solutions of 0.05 μg L^−1^ Cd(II), 0.15 μg L^−1^ Cu(II) and 0.5 μg L^−1^ Pb(II) at 0.01 mol L^−1^ HNO_3_.

#### 2.2.1. Effect of Chelating Agent and Sample Acidity

In this work, ammonium pyrrolidine dithiocarbamate (APDC) was used as chelating agent due to its well-known ability to form strong hydrophobic complexes in acidic solutions. Different concentrations (i.e., 0.01–0.5% *m*/*v*) of APDC solution were investigated to provide sufficient metal complexation and reduced chemical consumption. It was observed that an increase in the concentration up to 0.05% *m*/*v* had a positive effect, while any further increase in the concentration did not result in any additional benefits. However, since other potentially co-existing metals can consume the chelating reagent for metal complex formation, APDC was used in an excess concentration of 0.1% *m*/*v*.

The effect of sample acidity has been thoroughly investigated in earlier studies and it is evident that a stable metal complexation is observed using a nitric acid concentration of 1.0 × 10^−3^–1.0 × 10^−2^ mol L^−1^ that corresponds to sample pH values of 2–3 [14]. This parameter plays a crucial role for the efficient on-line formation and retention of the analyte-APDC complexes onto the surface of the sol-gel sorbent. In this case, a concentration of HNO_3_ of 0.01 mol L^−1^ was chosen to ensure the sufficient complexation.

#### 2.2.2. Effect of Loading Flow Rate and Preconcentration Time

The sample loading flow rate and the preconcentration time in automatic time-based systems are considered to be two critical parameters since they determine the sample volume, the analyte retention and the preconcentration time. In addition, they affect the velocity of the liquid inside the column and, thus, the contact time between the target analyte and the active groups of the sorbent which is crucial for their interaction. These parameters are directly associated with the sorption equilibration and retention of the metals on the sorptive phase of the FDSE medium [24]. The sample loading flow rate was investigated between 60 μL s^−1^ and 180 μL s^−1^ for a preconcentration time of 60 s, maintaining a sample/APDC flow rate ratio of ca. 15. As shown in Figure S4, an almost linear increase in method sensitivity was reported up to 150 μL s^−1^, demonstrating the efficient retention of the in situ-created metal complexes. At higher flow rates, a slight decrease in the response was observed. As a result, different loading flow rates can be employed in the on-line SI-FDSE procedure depending on the required sensitivity, the required sample throughput, and the sample availability. In this case, a sample loading flow rate of 150 μL s^−1^ was considered satisfactory for high method sensitivity, and it was finally adopted for further studies.

Subsequently, the preconcentration time was studied between 15 and 100 s. As it is presented in Figure S5, the increase in the absorbance for all analytes was practically proportional to the increase in preconcentration time. As a compromise between method high sensitivity and high sample throughput, a preconcentration time of 90 s was finally chosen.

#### 2.2.3. Effect of Eluent Type and Elution Flow Rate

In this study, MIBK was chosen as an eluent. This solvent has been proven to be a powerful eluent compared to organic solvents with lower polarity due to the limited dispersion of the eluted analyte and the efficient desorption of the retained complexes from the sorbent [25]. The elution flow rate of MIBK within the SI-FDSE system was investigated between 5 and 20 μL s^−1^. No significant response variations were observed within the examined range. Thus, an elution flow rate of 10 μL s^−1^ was chosen to ensure sufficient interaction between the adsorbed analytes and the MIBK.

#### 2.2.4. Effect of Sample Volume Injected in Graphite Furnace

In order to achieve good method sensitivity, the effect of different sample volumes injected in the ETAAS system were studied. This parameter determines the amount of analyte that is finally analyzed. In this study, the first portion of the eluent zone was injected in the ETAAS system, since it is expected to contain the desorbed analytes at higher concentration in comparison with other parts along the zone of the eluent. For this purpose, different quantities between 25 and 45 μL were studied. Sufficient method sensitivity was obtained using 35 μL of eluent and, thus, this value was finally chosen.

#### 2.2.5. Effect of Sample Ethanol Content

**Figure 2:**
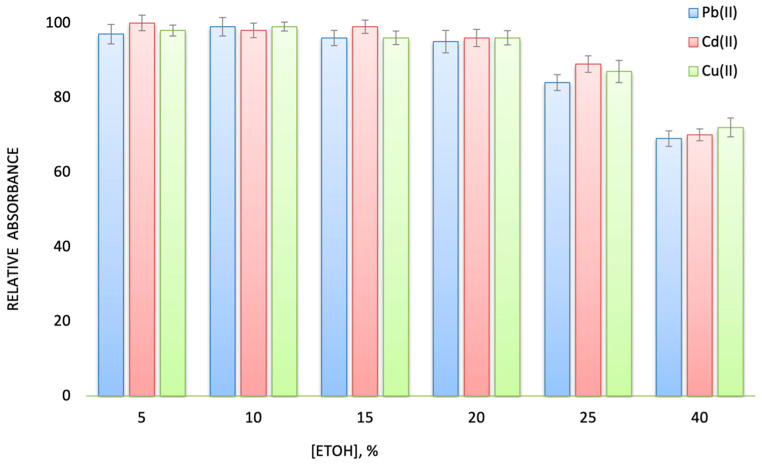
Effect of ethanol content (%) on the sensitivity of 0.05 μg L^−1^ Cd(II), 0.15 μg L^−1^ Cu(II), and 0.5 μg L^−1^ Pb(II). All parameters as presented in the operational sequence of the SI-FDSE-ETAAS method. The error bars were calculated based on standard deviation (±1 s).

The effect of ethanol concentration on the absorbance was studied between 5% and 40% *v*/*v* ethanol in water. As shown in Figure 2, absorbance for all analytes was constant at ethanol contents varying between 5 and 20% *v*/*v*, while a slight decrease was observed at higher ethanol content. This observation is in accordance with other studies about alcoholic spirit drinks, in which sample dilution is recommended since high ethanol contents reduce the retention of the target analytes due to the disruption of the interaction between them and the active groups of the sorptive phase [26,27]. Thus, to ensure the efficient retention of the metal, the distilled spirit drinks should be at least 1:1 (*v*/*v*) diluted with water prior to SI-FDSE procedure.

### 2.3. Figures of Merit

Table 1 presents the analytical characteristics of the developed SI-FDSE-ETAAS protocol. As it can be observed, good linearity was obtained for all analytes within the studied linear range. The utilization of a preconcentration time of 60 s resulted in a high sample throughput of 24 samples h^−1^. The enhancement factors for the target analytes were calculated as the ratio of the slope of the calibration curve for each metal after the on-line column preconcentration procedure versus the slope of the calibration curve for the same analyte without preconcentration (direct injection of 35 μL aqueous standard solution into the graphite furnace). The slopes of the batch ETAAS method were for 0.0605, 0.0052 and 0.0030 μg L^−1^ for Cd(II), Cu(II) and Pb(II), respectively.

Under optimum extraction conditions, the enhancement factors were found to be 38, 120 and 85 for Cd(II), Cu(II) and Pb(II), respectively. The limits of detection (LODs) and the limits of quantification (LOQs) were calculated according to the recommendation of the International Union of Pure and Applied Chemistry (IUPAC) following the 3 s and 10 s criteria, respectively.

The LOD values of the SI-FDSE-ETAAS method were 1.9 ng L^−1^ for Cd(II), 7.1 ng L^−1^ for Cu(II) and 17.3 ng L^−1^ for Pb(II). Moreover, the LOQ values were 6.4 ng L^−1^ for Cd(II), 23.6 ng L^−1^ for Cu(II) and 57.8 ng L^−1^ for Pb(II), demonstrating the applicability of the proposed method for trace metal analysis of distilled spirit drinks. The precision of the SI-FDSE-ETAAS method was calculated in terms of relative standard deviation (RSD) of ten repeated measurements of spiked distilled spirit drink samples containing 0.02 μg L^−1^ of Cd(II), 0.10 μg L^−1^ of Cu(II) and 0.25 μg L^−1^ of Pb(II). For all analytes, the RSD values were lower than 2.9%, indicating good precision.

The accuracy of the SI-FDSE-ETAAS method was evaluated by analyzing three different certified reference materials, i.e., NIST CRM 1643e (trace elements in water), IAEA-433 (marine sediment), and BCR 278-R (mussel tissue). Student’s *t*-test (probability level of 95%) was conducted for the examination of the significance of the differences that occurred between the certified and the experimentally calculated values. As shown in Table 2, in all cases the *t_exp_* values were lower than the *t_crit_*_, 95%_ = 4.30, showing no statistical differences and good method accuracy.

### 2.4. Interferences Studies

For a complete evaluate of the herein-developed analytical method, the effect of potentially interfering ions on the extraction performance was studied. For this purpose, a standard solution containing 0.02 μg L^−1^ of Cd(II), 0.10 μg L^−1^ of Cu(II), 0.20 μg L^−1^ of Pb(II) and different quantities of individual potential interferents were used, while the criterion of ≥5% of the analyte response was used to evaluate the existence of interferences. The results showed that the proposed SI-FDSE-ETAAS method can tolerate Al(III), Co(II), Fe(III), Mn(II), Ni(II) and Zn(II) at concentrations up to 10 mg L^−1^, Hg(II) at concentrations up to 2.5 mg L^−1^ and Ca(II), Mg(II), Ba(II), Na(I), K(I), Br^−^, Cl^−^, I^−^, SO_4_^2−^, NO_3_^−^ at concentrations up to 1000 mg L^−1^. As a result, the tolerance ratio for toxic metals was higher than 12,500 for all cases.

### 2.5. Real Sample Analysis

The proposed SI-FDSE-ETAAS method was utilized for the elemental assessment of different distilled spirit drinks. Prior to the analysis, a 1:1 (*v*/*v*) dilution of the samples with water was performed. For the accuracy evaluation, spiked solutions at appropriate concentration levels were also analyzed. The results of real sample analysis are shown in Table 3. Cadmium, copper and lead concentration levels ranged up to 0.122 μg L^−1^, up to 22.6 μg L^−1^, and up to 101.5 μg L^−1^, respectively. Moreover, the relative recoveries were 94.0–103.0%, demonstrating good method applicability.

The results for the real sample analysis were compared with the concentrations found in previous works. As shown in Table 4, similar concentrations levels were observed between the equivalent types of alcoholic beverages.

### 2.6. Comparison with Other Studies

The developed SI-FDSE-ETAAS method was compared with other previously reported on-line ETAAS methods and the results of the comparative study are summarized in Table 5.

As it can be observed, the proposed method resulted in enhanced sensitivity for all the target analytes. At the same time, the SI-FDSE-ETAAS method is associated with adequate sample consumption, fast kinetics, high enhancement factors and good method precision. It is noteworthy that the herein-reported packed microcolumn is also characterized by fabrication simplicity, negligible back pressure, and high reusability (at least 500 cycles), making it an ideal option for on-line ETAAS methodologies for routine analysis.

### 2.7. Evaluation of the Green Character of the Proposed Method

**Figure 3:**
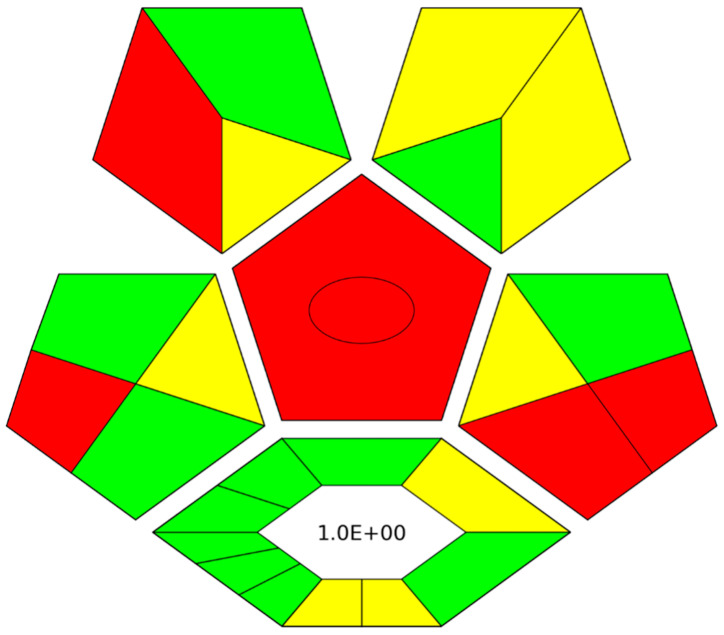
ComplexGAPI pictogram for the SI-FDSE-ETAAS method.

In order to evaluate the green character of the on-line SI-FDSE-ETAAS method, complexGAPI index was used [45]. This tool enables the evaluation of the main parameters used for the analytical determination (i.e., sample collection, sample storage, sample transfer, sample preparation, chemicals and reagents, instrumentation, and method type), as well as the synthetic procedure used for the preparation of novel extraction phases, stationary phases, solvents, etc. Thus, this index was selected to provide a complete assessment of the proposed method from the production of FDSE membranes to analytes determination by ETAAS. The obtained pictogram is shown in Figure 3. It can be observed that regarding the production of the novel sorptive phases most of the requirements of complexGAPI index are met (green color in the hexagon). The synthetical route is characterized by a low *E*-factor, thus supporting the green economy. At the same time, a high process yield is obtained, relatively mild conditions are used, and a low amount of waste is generated. Regarding the analytical part of the SI-FDSE-ETAAS protocol, micro-scale extraction is used resulting in reduced consumption of hazardous chemicals. All things considered; the proposed method exhibits a green character in compliance with the principles of Green Analytical Chemistry [46].

## 3. Materials and Methods

### 3.1. Reagents, Materials and Samples

Concentrated nitric acid (65%), methyl isobutyl ketone (MIBK) and stock standard solutions (1000 mg L^−1^) of Cd(II), Cu(II) and Pb(II) were purchased from Merck (Merck, Darmstadt, Germany). Ultra-pure water produced by a Milli-Q system, also by Merck (Merck, Darmstadt, Germany), was used throughout the study. Working solutions for the target analytes were prepared on a daily basis through appropriate dilution of the stock standards. Ammonium pyrrolidine dithiocarbamate (APDC) and sol-gel precursors, methyl trimethoxysilane (MTMS) and tetramethyl orthosilicate (TMOS), were purchased from Sigma Aldrich (St. Louis, MO, USA). Aqueous solutions of APDC were prepared daily at appropriate concentration levels. The polyester fabric substrate was purchased from Joanne Fabrics (Miami, FL, USA). Isopropanol, HCl, NH_4_OH, and methylene chloride were purchased from Fisher Scientific (Milwaukie, WI, USA). A single-layer graphene oxide dispersion in ethanol (5 mg mL^−1^) was purchased from ACS Material (Pasadena, CA, USA). The preparation of the coated polyester fabric membranes is described in the Supplementary Materials.

The proposed method was applied to the analysis of different types of alcoholic beverages (i.e., rum, vodka, gin, and tsipouro) with an ethanol content up to 40%. All samples were randomly purchased during April 2022 from the local market of Thessaloniki.

### 3.2. Instrumentation

A Perkin Elmer, Norwalk, Connecticut, USA (http://www.perkinelmer.com, accessed on 22 February 2023) model 5100 PC flame atomic absorption spectrometer with Zeeman-effect background correction equipped with AS-71 furnace auto-sampler was employed throughout study. Pyrolytically coated THGA graphite tubes (Perkin Elmer) with integrated L’vov platform were used. Argon 99.996% was applied as purge and protective gas. A hollow cathode lamp operated at 4 mA for Cd, a hollow cathode lamp operated at 30 mA for Cu, and an electrodeless discharge lamp operated at 10 W for Pb were used as light sources. The wavelength was set at 228.8 nm resonance line, at 324.7 nm and 283.3 nm for cadmium, copper and lead, respectively, while the monochromator spectral bandpass (slit) was set at 0.7 nm. Integrated absorbance (peak area) was used for signal evaluation throughout the study. The graphite furnace temperature/time program is presented in Table S1 (Supplementary Materials).

**Figure 4:**
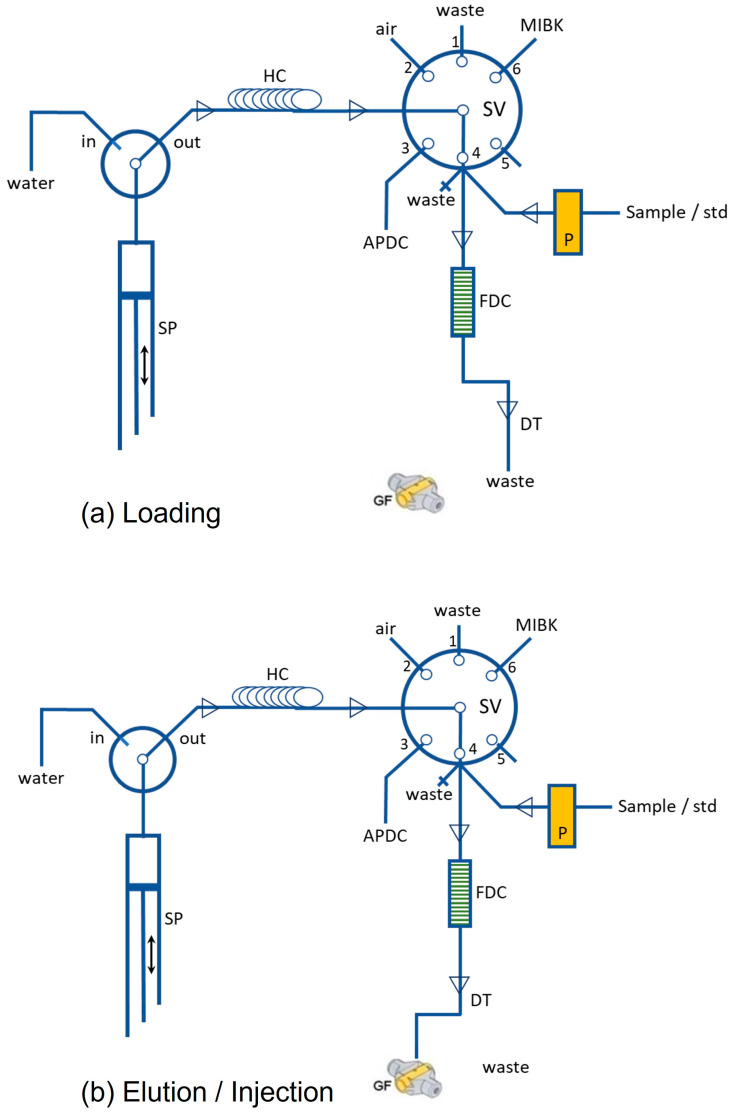
Schematic diagram of the proposed SI-FDSE-ETAAS manifold (**a**) loading, (**b**) elution/injection.

The online FDSE procedure took place utilizing a sequential injection (SI) system model FIALab^®^-3000 (FIAlab, Alitea, USA) consisting of a syringe pump (SP) with a glass barrel capacity of 1000 μL (Cavro, Sunnyvale, CA, USA), one six-port selection valve (SV), and one peristaltic pump (P) for sample or standard solution propulsion. The schematic diagram of the SI-FDSE-ETAAS procedure is depicted in Figure 4.

The SI system was controlled by a personal computer and the FIAlab application software for windows ν.5.9.245 (http://www.flowinjection.com, accessed on 22 February 2023). The complete system was commanded by the computer that controlled the SIA system.

For the characterization of the FDSE disks, a Philips XL30 Scanning Electron Microscope (Cambridge, MA, USA) equipped with an EDAX detector and an Agilent Cary 670 FTIR Spectrometer (Santa Clara, CA, USA) were used.

### 3.3. Fabrication of the FDSE Microcolumn

The FDSE disks were obtained by cutting the fabric material into 40 disks of 4.0 mm i.d. using an appropriate hollow punch tool. For the fabrication of the FDSE microcolumn, a 1000 μL disposable polypropylene syringe of 10.0 cm length and 4.0 mm i.d. was shortened to 1.5 cm and packed with the FDSE disks. Prior to its use, the freshly prepared FDSE microcolumn was flashed with a solution of 1.0 mol L^−1^ HNO_3_ solution, followed by rinsing with Milli-Q water to remove any undesired impurities from the surface of the FDSE disks. The microcolumn fabrication procedure is characterized by simplicity, and the obtained microcolumn can be easily repacked with new FDSE membranes when necessary. Moreover, no frits or glass wool is necessary to block the FDSE media at the upper and lower part of the syringe. Due to the easy permeation of the incoming flow, through the pores of the FDSE disks, negligible back-pressure was observed, while high flow rates could be utilized resulting in rapid extraction and high extraction efficiency. It is noteworthy that the prepared FDSE microcolumns were proven to be reusable for at least 500 sample loading/elution cycles.

### 3.4. On-Line FDSE Analytical Procedure

The herein-developed automatic on-line analytical SI-FDSE-ETAAS procedure was operated in 12 individual steps categorized under four main sequences *a–d*. Table 6 (Supplementary Materials) summarizes the operational sequences used for the determination of Cd(II), Cu(II) and Pb(II). Using the proposed program, the sample analysis time was 150 s, and the sampling frequency (*f*) was 24 h^−1^. For all instances, five replicates were performed. The detailed sequences are given in the Supplementary Materials.

## 4. Conclusions

A novel SI-FDSE on-line automatic system was proposed as a front-end to ETAAS for the monitoring of trace amounts of toxic elements in distilled spirit drinks. The novel manifold showed ease in fabrication, low cost, fast extraction kinetics, high extraction performance, high reusability and low back-pressure. Moreover, the developed analytical method exhibited good accuracy, precision, linearity and low LOD and LOQ values. The proposed SI-FDSE-ETAAS method was successfully employed for the determination of Cd(II), Cu(II) and Pb(II) in a wide range of alcoholic beverages. The proposed system could be also used for the monitoring of other toxic elements with similar complexing reagents in other beverage samples (e.g., beer, wine). This system could be also expanded to extract other analytes (e.g., nickel, cobalt, etc.). For this purpose, different sol-gel-coated FDSE media can also be examined to ensure optimum extraction performance for the new analytes. Finally, the herein-developed SI-FDSE platform could also be used as a front-end to inductively coupled plasma atomic emission spectrometry after back-extraction, aiming to perform multi-elemental analysis.

## Figures and Tables

**Table 1:** Figures of merit for the developed SI-FDSE-ETAAS method.

Parameter	Cd(II)	Cu(II)	Pb(II)
Slope, *S* (μg L^−1^)	2.3000 ± 0.1004	0.6272 ± 0.0256	0.2568 ± 0.0078
Intercept, *i*	0.0030 ± 0.0093	0.0022 ± 0.0100	0.0033 ± 0.0055
Linear range (μg L^−1^)	0.0064–0.160	0.0236–0.70	0.0578–1.5
Correlation coefficient, *r*^2^	0.9985	0.9987	0.9993
LOD (ng L^−1^)	1.9	7.1	17.3
LOQ (ng L^−1^)	6.4	23.6	57.8
RSD (%)	2.4 (0.02 μg L^−1^)	2.2 (0.10 μg L^−1^)	2.9 (0.25 μg L^−1^)
Preconcentration time (s)	90	90	90
Sample throughput (h^−1^)	24	24	24
Enhancement factor	38	120	85
Sample consumption (mL)	13.5	13.5	13.5

**Table 2:** Determination of Cd(II), Cr(VI) and Pb(II) in certified reference materials Mean value ± standard deviation (*n* = 3), *t*_*crit*, 95%_ = 4.30.

Certified Reference Material	Cd	Cu	Pb
CRM 1643e (Trace Element Water)			
Certified value (μg L^−1^)	6.568 ± 0.073	22.76 ± 0.31	19.63 ± 0.21
Found (μg L^−1^)	6.35 ± 0.29	22.26 ± 0.66	18.9 ± 1.12
Relative Error	−3.3	−2.2	−3.7
*t*_*exp*_	1.302	1.312	1.129
IAEA-433 (Marine sediment)			
Certified value (μg L^−1^)	0.153 ± 0.033	30.8 ± 2.6	26.0 ± 2.7
Found (μg L^−1^)	0.149 ± 0.015	29.5 ± 1.2	26.4 ± 1.8
Relative Error	−2.6	−4.2	1.5
*t*_*exp*_	0.462	1.876	−0.385
BCR 278-R (Mussel tissue)			
Certified value (μg L^−1^)	0.348 ± 0.007	9.45 ± 0.13	2.00 ± 0.04
Found (μg L^−1^)	0.33 ± 0.02	9.32 ± 0.35	1.90 ± 0.09
Relative Error	−5.2	−1.4	−5.0
*t*_*exp*_	1.559	0.643	1.925

**Table 3:** Analytical results for Cd, Cu, and Pb in distilled spirit drinks samples (*n* = 3).

Sample		Cd			Cu ^1^				Pb			
	Added (μg L^−1^)	Found (μg L^−1^)	Recovery (%)	*t*_*exp*_	Added (μg L^−1^)	Found (μg L^−1^)	Recovery (%)	*t*_*exp*_	Added (μg L^−1^)	Found (μg L^−1^)	Recovery (%)	*t*_*exp*_
Rum-1	-	0.090 ± 0.007	-	-	-	6.20 ± 0.90	-	-	-	2.12 ± 0.15	-	-
	0.200	0.284 ± 0.017	97.0	0.611	0.500	6.67 ± 0.50	94.0	0.104	0.500	2.60 ± 0.18	96.0	0.192
Rum-2	-	<LOD	-	-	-	7.90 ± 0.60	-	-	-	1.48 ± 0.10	-	-
	0.200	0.190 ± 0.014	95.0	1.237	0.500	8.41 ± 0.80	102.0	−0.022	0.500	2.000 ± 0.11	104.0	−0.315
Vodka-1	-	<LOD	-	-	-	10.30 ± 0.90	-	-	-	<LOD	-	-
	0.200	0.188 ± 0.012	94.0	1.732	0.500	10.78 ± 0.80	96.0	0.043	0.500	0.488 ± 0.661	97.6	0.031
Vodka-2	-	<LOD	-	-	-	4.60 ± 0.30	-	-	-	0.220 ± 0.011	-	-
	0.200	0.192 ± 0.015	96.0	0.924	0.500	5.09 ± 0.30	98.0	0.058	0.500	0.700 ± 0.032	96.0	1.155
Gin-1	-	<LOD	-	-	-	5.90 ± 0.20	-	-	-	<LOD	-	-
	0.200	0.210 ± 0.017	105.0	−1.019	0.500	6.38 ± 0.40	96.0	0.087	0.500	0.475 ± 0.033	95.0	1.443
Gin-2	-	0.120 ± 0.009	-	-	-	9.20 ± 0.70	-	-	-	1.50 ± 0.12	-	-
	0.200	0.325 ± 0.030	102.5	−0.289	0.500	9.73 ± 0.60	106.0	−0.087	0.500	1.97 ± 0.11	94.0	0.472
Tsipouro-1	-	0.100 ± 0.009	-	-	-	12.30 ± 1.00	-	-	-	30.2 ± 1.6 ^1^	-	-
	0.200	0.293 ± 0.020	96.5	0.606	0.500	12.83 ± 1.10	106.0	−0.047	5.0	35.1 ± 1.8	98.0	0.096
Tsipouro-2	-	<LOD	-	-	-	12.10 ± 0.90	-	-	-	25.4 ± 5.0 ^1^	-	-
	0.200	0.205 ± 0.015	102.5	−0.577		12.59 ±0.80	98.0	0.022	5.0	30.5 ± 1.7	101.0	−0.551

^1^ 1:25 *v*/*v* dilution.

**Table 4:** Comparison of the elemental concentration levels (μg L^−1^) in distilled spirit drinks with other studies.

Sample	Cd	Cu	Pb	Ref.
Gin	0.08–1.12	-	-	[28]
Rum	ND–0.70	-	-	
Rum	3.0–30	ND–640	50–220	[29]
Gin	10–30	ND–70	100–130	
Vodka	10–30	ND–90	80–380	
Rum	-	3–45	23–65	[30]
Fruit spirits	ND–6.6	-	-	[31]
Gin	-	-	ND–35.70	[32]
Rum	-	-	ND–70.00	
Pomace brandy (Tsikoudia)	ND–1	55–105,000	<1–1200	[33]
Rum	ND	ND–276,000	ND–16	[34]
Rum	-	ND–400	-	[35]
Rum	-	-	1.25	[36]
Rum, vodka, gin, tsipouro	ND–0.120	4.60–12.30	ND–30.2	This work

ND: Not detected.

**Table 5:** Comparison of the developed SI-FDSE-ETAAS method with previously reported on-line ETAAS methods.

Analyte	On-Line Procedure	P.T. (s)	S.C. (mL)	E.F.	RSD (%)	LOD (μg L^−1^)	Ref.
Cd(II)/ Pb(II)	SI-DLLME	90	8.1	34/ 80	4.1/ 3.8	0.002/ 0.01	[37]
Cd(II)	SI-SPE based on octadecylsilane functionalized maghemite particles	250	5.0	19	3.9	0.003	[38]
Cd(II)	SI–bead injection–lab-on-valve platform equipped with a microcolumn packed with PTFE beads	52	1.25	17.2	4.3	0.015	[39]
Cu(II)	SI-SPE based on silk fibroin sorbent	90	0.9	27.3	2.2	0.008	[40]
Cd(II)	SI-single-drop micro-extraction	600	15	10	3.9	0.01	[41]
Pb(II)	FI-SPE based on a chelating resin immobilized on aminopropyl-controlled pore glass	90	3.3	20.5	3.2	0.012	[42]
Cd(II)	SI-solvent extraction-back extraction	130	13.0	21.4	0.4	0.0027	[43]
Cu(II)	SI-SPE based on PTFE-beads-packed column	17	1.0	20	1.8	0.015	[44]
Cd(II)/ Cu(II)/ Pb(II)	SI-FDSE	90	13.5	38/ 120/ 85	2.4/ 2.2/ 2.9	0.0019/ 0.0071/ 0.0173	This work

**Table 6:** Operational sequence of the SI-FDSE-ETAAS method for metal determination.

Step ^a^	V Position	SV Position	SP Flow Rate (μL s^−1^)	SP Operation	SP Volume (μL)	P Operation	Commentary
1	IN	2	80	Aspirate	50	OFF (*)	Water into SP
2	OUT	2	5	Aspirate	10	OFF	Air segment into HC
3	OUT	3	80	Aspirate	910	OFF	APDC into HC
4	OUT	4	10	Dispense	900	ON (*)	Sample loading, preconcentration for 90 s
5	OUT	1	80	Dispense	70	OFF	Emptying the SP
6	IN	1	80	Aspirate	300	OFF	Water into SP
7	OUT	6	10	Aspirate	500	OFF	MIBK into HC
8	OUT	4	5	Dispense	280	OFF	Elution– Transportation of eluent up to the exit of DT
9							DT into GF
10	OUT	4	5	Dispense	35	OFF	35 μL of MIBK into GF
11							DT back to waste. Starting the ETAAS program/measuring
12	OUT	4	50	Dispense	485	OFF	Cleaning of micro-column, Emptying the SP

^a^ Sample propulsion with flow rate, (150 μL s^−1^); SP, syringe pump; V, valve of SP; SV, multi-position selection valve; P, peristaltic pump; GF, graphite furnace of ETAAS. * ON means that the pump is operating and OFF means that the pump is not operating.

## Data Availability

Not applicable.

## Supplementary Materials

The following supporting information can be downloaded at: https://www.mdpi.com/article/10.3390/molecules28052103/s1, Fourier Transform Infra-Red Spectroscopy (FT-IR), Preparation of the of the coated polyester fabric membranes [47,48]. Main sequences of the on-line procedure, Figure S1: FT-IR spectra of (a) pristine graphene oxide; (b) sol-gel graphene oxide-coated polyester fabric phase sorptive extraction membrane; Figure S2: FT-IR spectra of uncoated polyester substrate; Figure S3: FT-IR spectra of methyl trimethoxysilane; Figure S4: Effect of sample flow rate on the extraction efficiency of 0.05 μg L^−1^, Cd(II), 0.15 μg L^−1^ Cu(II), and 0.5 μg L^−1^ Pb(II). All parameters as presented in Table 6. The error bars were calculated based on standard deviation (±1 s); Figure S5: Effect of loading time on the extraction efficiency of 0.05 μg L^−1^ Cd(II), 0.15 μg L^−1^ Cu(II), and 0.5 μg L^−1^ Pb(II). All parameters as presented in Table 6. The error bars were calculated based on standard deviation (±1 s).; Table S1: Graphite furnace temperature/time program for Cd, Cu and Pb determination in 35 μL of MIBK.

## Sample Availability

Samples of the compounds/analytes are available from the authors.
