# Supplementary material for: Sol-Gel Graphene Oxide-Coated Fabric Disks as Sorbents for the Automatic Sequential-Injection Column Preconcentration for Toxic Metal Determination in Distilled Spirit Drinks
Source: Molecules. 2023 Feb 23;28(5):2103. doi: 10.3390/molecules28052103 (PMC10004266; doi:10.3390/molecules28052103)
Supplement: Supplementary file 1 [file molecules-28-02103-s001.zip › molecules-2224604-supplementary.pdf]

Supplementary Material

Sol-Gel Graphene Oxide-Coated Fabric Disks as Sorbents for the Automatic Sequential-Injection Column Preconcentration for Toxic Metal Determination in Distilled Spirit Drinks

Natalia Manousi [1], Abuzar Kabir [2], Kenneth G. Furton [2] and Aristidis Anthemidis [2,*]

[1] Laboratory of Analytical Chemistry, Department of Chemistry, Aristotle University of Thessaloniki, 54124 Thessaloniki, Greece

[2] International Forensic Research Institute, Department of Chemistry and Biochemistry, Florida International University, Miami, FL 33131, USA

* Correspondence: anthemid@chem.auth.gr

Fourier Transform Infra-Red Spectroscopy (FT-IR)

The FT-IR spectra of (a) pristine graphene oxide particles; (b) sol-gel graphene oxide coated polyester FPSE membrane are presented in Figure S1. FT-IR spectra of uncoated polyester fabric and methyl trimethoxysilane are presented in Supplementary Figures S3, and S4, respectively. Noteworthy features of pristine graphene oxide FT-IR spectra include bands at 1065 cm^{-1}, 1635 cm^{-1} and 3305 cm^{-1} that represent C-O-C, C-C, and O-H stretching vibrations of –COOH, respectively [47]. The major features of uncoated polyester fabric FT-IR spectra (Figure S4) include weak C-H stretching at 2963 cm^{-1}, anhydride groups at 1968 cm^{-1}, C=O symmetric stretching of the carbonyl groups at 1712 cm^{-1}, out-of-plane bending in the terminal carboxylic groups in the polyester chain at 1016 cm^{-1}, C-C out-of-plane bending vibrations of the benzene ring of the polymer at 865 cm^{-1} and C-H bending at 718 cm^{-1} [48]. Important features of MTMS spectra (Figure S5) include characteristic Si-O-C-H bond at 2947 cm^{-1} and Si-OCH_3 bands at 2839 cm^{-1} and 1077 cm^{-1}. The bands at 1264 cm^{-1} and 789 cm^{-1} represent the vibrations of –CH_3 pendant group of the precursor. Many features of MTMS FT-IR spectra can be seen in the sol-gel graphene oxide FT-IR spectra, however, no characteristic feature of graphene oxide FT-IR spectra can be seen in the sol-gel graphene oxide FT-IR spectra. Very low relative abundance of graphene oxide in the sol-gel graphene oxide sorbent coating may explain the origin of this observation.

Preparation of the of the Coated Polyester Fabric Membranes

To prepare the sol-gel coating of the polyester substrate surface, 10 mL of ethanolic dispersion of graphene oxide (5 mg mL^{-1}) was further diluted in ethanol to obtain 1 mg mL^{-1} graphene oxide dispersion in ethanol. A 50 cm^2 pretreated polyester substrate was immersed into 200 mL of ethanolic dispersion of graphene oxide and was kept immersed under sonication for 30 min. Subsequently, the polyester substrate retaining the nanoparticles by physical adsorption was transferred to into the reaction vessel containing the sol solution. The sol solution was prepared by sequential addition of 10 mL sol-gel precursor MTMS, 10 mL methylene chloride, 10 mL acetone, and 4 mL aqueous trifluoroacetic acid (95% in water) into a large centrifuge. After vortex mixing for 3 min, centrifugation for 5 min, and sonication for 2 min, the clear supernatant part of the sol solution was finally transferred to a clean 3 oz. amber colored glass reaction bottle. The polyester substrate was kept submerged into the sol solution for 4 h to form the sol–gel coating around the micro fibrils of the substrate and eventually encapsulates sorbent particles within the sol-gel network.

Following the completion of the residence time of the polyester substrate into the sol solution, the coated substrate was removed from the sol solution and was kept in the desiccator overnight for the evaporation of the solvent and the of aging of the sol–gel coating. The coated FPSE membrane was subsequently rinsed with methylene chloride: acetone

(50:50; v/v) under sonication for 30 min to remove unreacted and un-bonded residual sol solution ingredients from the coated surface. The FPSE membrane was then air dried for 1 h and stored in airtight containers prevented accumulation of unwanted analytes from the environment.

Main Sequences of the On-Line Procedure

Sequence a (steps 1–4): Initially, the valve of SP was in "IN position" and water was aspirated into the SP. Subsequently, the valve of SP was switched to "OUT position" and a segment of air was aspirated into the HC, followed by 0.1% w/v APDC solution. The stream of the metal chelating reagent was dispensed through the FDSE microcolumn simultaneously with the sample solution (sample propulsion with flow rate, (150 μL s^{-1}) and the metal complexes were retained by the sol-gel sorbent.

Sequence b (steps 5–8): Following the retention of the target analytes, the SP was emptied and re-filled with water. An aliquot of 500 μL of MIBK was aspirated into the HC and it was propelled through the FDSE microcolumn at a flow rate of 5 μL s^{-1} for the elution of the metal complexes. Accordingly, the eluent was transferred up to the exit of DT.

Sequence c (steps 9–11): The eluent containing the desorbed analytes was delivered into the GF for metal determination by ETAAS.

Sequence d (steps 12): The microcolumn was cleaned and the SP was emptied aiming to prepare the manifold for the next retention/elution cycle.

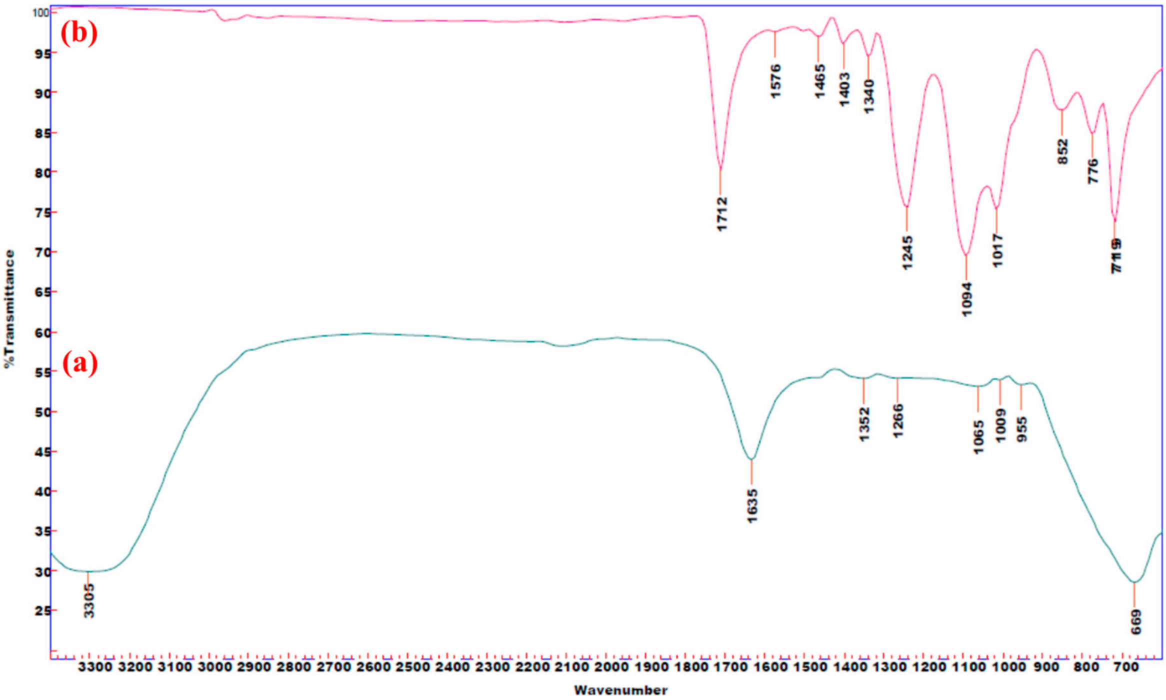

Figure S1. FT-IR spectra of (**a**) pristine graphene oxide; (**b**) sol-gel graphene oxide coated polyester fabric phase sorptive extraction membrane.

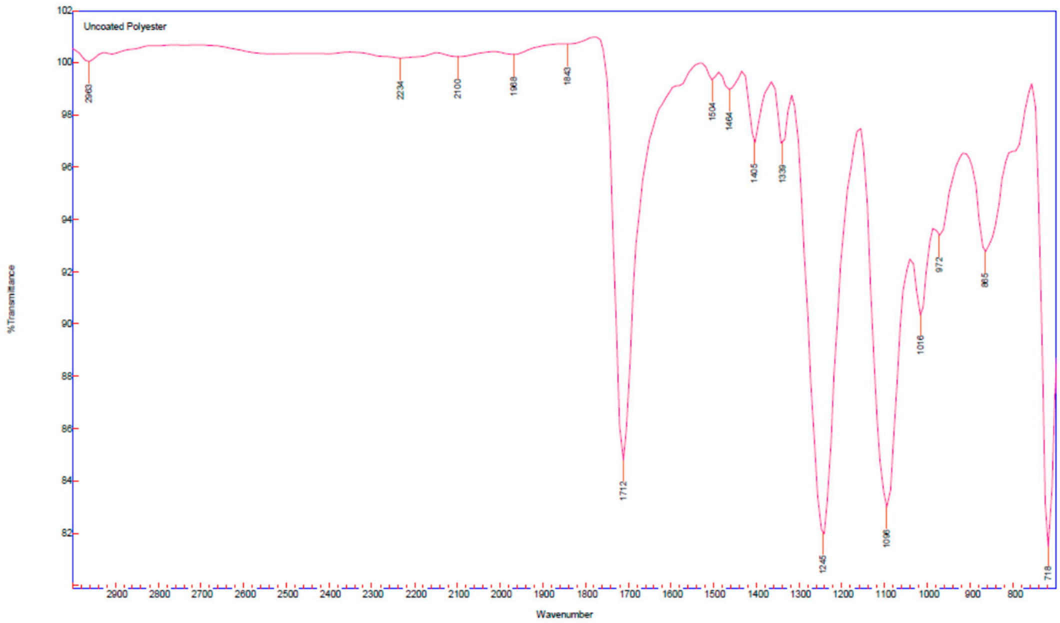

Figure S2. FT-IR spectra of uncoated polyester substrate.

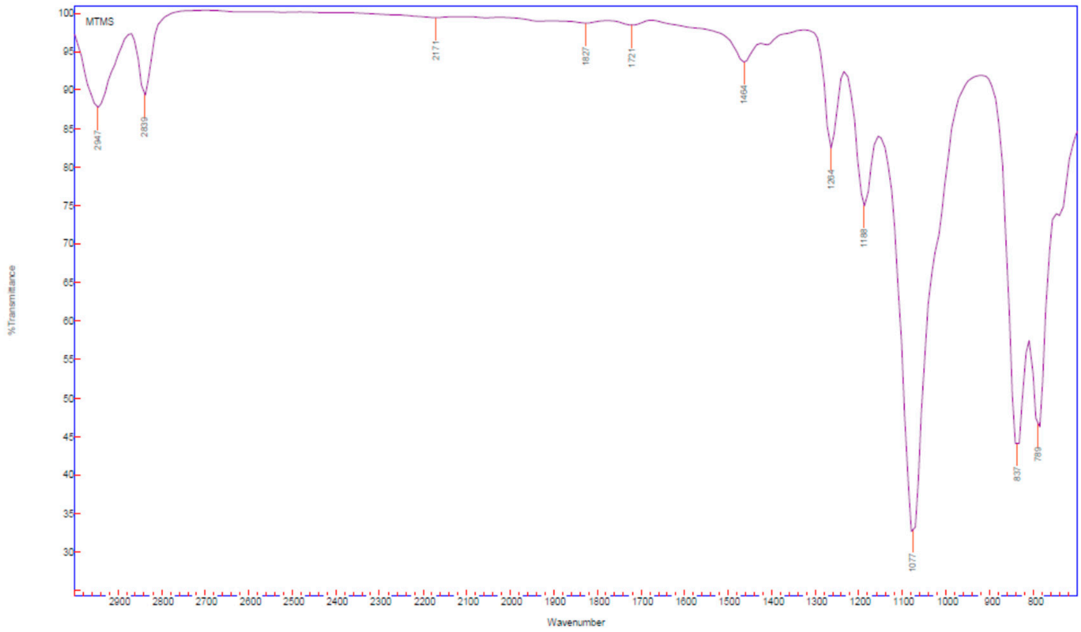

Figure S3. FT-IR spectra of methyl trimethoxysilane.

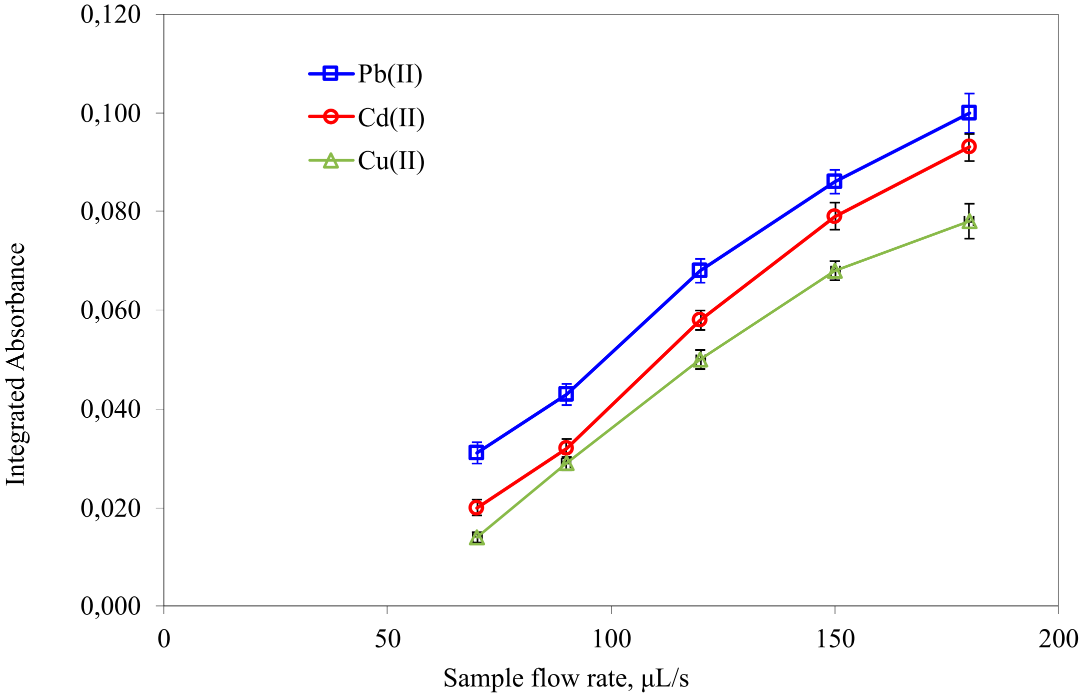

Figure S4. Effect of sample flow rate on the extraction efficiency of 0.05 µg L^{-1} Cd(II), 0.15 µg L^{-1} Cu(II), and 0.5 µg L^{-1} Pb(II). All parameters as presented in Table 6. The error bars were calculated based on standard deviation (± 1 *s*).

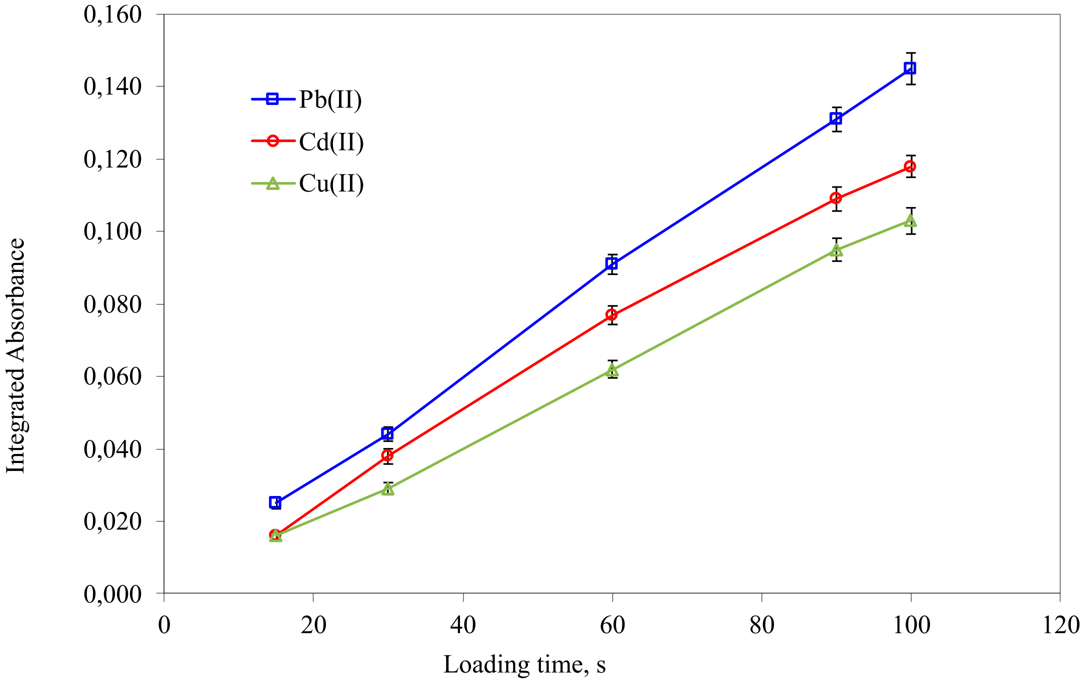

Figure S5. Effect of loading time on the extraction efficiency of 0.05 µg L^{-1} Cd(II), 0.15 µg L^{-1} Cu(II), and 0.5 µg L^{-1} Pb(II). All parameters as presented in Table 6. The error bars were calculated based on standard deviation (± 1 *s*).

Table S1. Graphite furnace temperature/time program for Cd, Cu and Pb determination in 35 μL of MIBK.

Step	Cd	Cu	Pb
Preheating Temp. (°C)	90	90	90
Ramp time (s)	10	10	10
Hold time (s)	10	10	10
Ar gas flow rate (mL min^{-1})	250	250	250
Drying Temp. (°C)	110	110	110
Ramp time (s)	5	5	5
Hold time (s)	20	20	20
Ar gas flow rate (mL min^{-1})	250	250	250
Pyrolysis Temp. (°C)	400	700	700
Ramp time (s)	20	10	10
Hold time (s)	20	20	20
Ar gas flow rate (mL min^{-1})	250	250	250
Atomization Temp. (°C)	2200	2300	2300
Ramp time (s)	0	0	0
Hold time (s)	5	5	5
Ar gas flow rate (mL min^{-1})	0	0	0
Cleaning Temp. (°C)	2400	2400	2400
Ramp time (s)	1	1	1
Hold time (s)	2	2	2
Ar gas flow rate (mL min^{-1})	250	250	250

References

1. Samanidou, V., Filippou, O., Marinou, E., Kabir, A., Furton, K.G. Sol–gel-graphene-based fabric-phase sorptive extraction for cow and human breast milk sample cleanup for screening bisphenol A and residual dental restorative material before analysis by HPLC with diode array detection. *J. Sep. Sci.* **2017**, *40*, 2612–2619.
2. Parvinzadeh, M., Ebrahimi, I. Influence of atmospheric-air plasma on the coating of a nonionic lubricating agent on polyester fiber. *Radiat. Eff. Defects Solids* **2011**, *166*, 408–416.
